# Glia-Pinealocyte Network: The Paracrine Modulation of Melatonin Synthesis by Tumor Necrosis Factor (TNF)

**DOI:** 10.1371/journal.pone.0040142

**Published:** 2012-07-02

**Authors:** Sanseray da Silveira Cruz-Machado, Luciana Pinato, Eduardo Koji Tamura, Cláudia Emanuele Carvalho-Sousa, Regina P. Markus

**Affiliations:** 1 Laboratory of Chronopharmacology, Institute of Biosciences, University of São Paulo, São Paulo, SP, Brazil; 2 Department of Speech-Language and Hearing Therapy, Universidade Estadual Paulista (UNESP), Marília, São Paulo, Brazil; Virginia Commonwealth University, United States of America

## Abstract

The pineal gland, a circumventricular organ, plays an integrative role in defense responses. The injury-induced suppression of the pineal gland hormone, melatonin, which is triggered by darkness, allows the mounting of innate immune responses. We have previously shown that cultured pineal glands, which express toll-like receptor 4 (TLR4) and tumor necrosis factor receptor 1 (TNFR1), produce TNF when challenged with lipopolysaccharide (LPS). Here our aim was to evaluate which cells present in the pineal gland, astrocytes, microglia or pinealocytes produced TNF, in order to understand the interaction between pineal activity, melatonin production and immune function. Cultured pineal glands or pinealocytes were stimulated with LPS. TNF content was measured using an enzyme-linked immunosorbent assay. TLR4 and TNFR1 expression were analyzed by confocal microscopy. Microglial morphology was analyzed by immunohistochemistry. In the present study, we show that although the main cell types of the pineal gland (pinealocytes, astrocytes and microglia) express TLR4, the production of TNF induced by LPS is mediated by microglia. This effect is due to activation of the nuclear factor kappa B (NF-kB) pathway. In addition, we observed that LPS activates microglia and modulates the expression of TNFR1 in pinealocytes. As TNF has been shown to amplify and prolong inflammatory responses, its production by pineal microglia suggests a glia-pinealocyte network that regulates melatonin output. The current study demonstrates the molecular and cellular basis for understanding how melatonin synthesis is regulated during an innate immune response, thus our results reinforce the role of the pineal gland as sensor of immune status.

## Introduction

The pineal gland, a circumventricular organ, plays an integrative role in the neuro-endocrine-immune response [1]–[3]. In mammals, the major cellular component of the pineal gland is the pinealocyte. In rats, this cell type comprises around 90% of the gland and the remainder consists mainly of glial cells and nerve fibers [4]. The pinealocytes are responsible for synthesizing melatonin, the darkness hormone, while the functions of astrocytes and microglia in the pineal gland are currently unclear.

The pineal gland only synthesizes melatonin at night due to nocturnal gene transcription and an increase in the activity of the enzyme arylalkylamine-N-acetyltransferase (AA-NAT) induced by noradrenaline activation of beta-adrenoceptors [5]–[7]. The daily rhythm of melatonin is driven by pineal production. Gastrointestinal cells, immunocompetent cells, and astrocytes also synthesize melatonin, which plays a local role related to tissue protection [8]–[16].

The pineal and extrapineal sources of melatonin production have been linked to independent physiological or pathophysiologycal contexts. Pineal melatonin, the darkness hormone, is responsible for effects dependent on low levels (pM range) of the indolamine, whereas extrapineal melatonin acts as a paracrine or autocrine mediator reaching higher concentrations (mM range) [1]. ‘Chronobiotic’ levels of melatonin inhibit both the rolling and adherence of leukocytes to the endothelial layer, and reduce vascular permeability [17], [18], avoiding unnecessary innate immune responses. In order to develop a full innate immune response, nocturnal pineal melatonin synthesis is suppressed both in birds and mammals [1], [19], [20]. In addition, immune-competent cells present in inflamed tissues are stimulated to synthesize melatonin, which acts in a paracrine manner as an anti-inflammatory mediator [1], [9], [11], [16]. This new approach in interpreting data regarding the two main functions of melatonin suggests that it is necessary to stop translating environmental lighting changes to the body, in order to allow the induction of inflammatory responses, which are essential for proper healing.

Prolonged disruption of chronobiotic pineal function correlates with several pathologies. An understanding of the mechanistic basis involved in the suppression of pineal melatonin synthesis during induction of innate immune responses will provide new insights in the relationship between the temporal organization of physiological functions and the genesis and maintenance of the pathological status.

Recently we have reported that the rat pineal gland responds to lipopolysaccharide (LPS), an endotoxin found in the outer membrane of gram-negative bacteria. This pathogen-associated molecular pattern triggers the activation of the transcription factor nuclear factor kappa B (NF-kB). In the pineal gland, LPS induces a rapid and transient activation of this pathway that can suppress melatonin synthesis [21]. In addition, LPS also triggers TNF production *in vitro* [21]. TNF inhibits melatonin synthesis by blocking AA-NAT gene transcription in cultured rat pineal glands [22]. High levels of TNF correlate with the inhibition of nocturnal melatonin in humans [11], [12], [23]. Furthermore, TNF is one of the first pro-inflammatory agents produced at the beginning of an immune response and amplifies and prolongs inflammatory responses. In the rat pineal gland, TNF is recognized by TNFR1 expressed on astrocytes, microglia and pinealocytes [24]. However, the source of TNF production in cultured pineal glands is currently unknown.

The aim of the present study was to elucidate the mechanism of action of LPS in the pineal gland, by evaluating its effect on different cell types. Therefore, we evaluated the localization of LPS receptors in different pineal gland cell types, and the downstream activation of NF-kB nuclear translocation which results in TNF synthesis. In addition, we analyzed the mechanism involved in the control of melatonin synthesis by LPS. Our data show that TLR4 is expressed on microglia, astrocytes and pinealocytes. In addition, we demonstrated that LPS induced microglial activation and TNF production, which may interact with TNFR1 that is up-regulated on pinealocytes. Our data also highlight the relevance of the NF-kB pathway in regulating melatonin synthesis. In this context, the production of TNF by microglia indicates an additional mechanism for controlling melatonin output and suggests a glia-pinealocyte regulatory network during inflammation. Therefore, we reinforce the hypothesis that the pineal gland is a sensor of immune status and provide a molecular basis that explains how melatonin synthesis is suppressed, as observed during clinical and experimental inflammatory conditions.

## Methods

### Animals

Pre-pubertal male and female Wistar rats (6 weeks, 84 animals) from the animal facility of the Department of Physiology (IB-USP, São Paulo, Brazil), were kept under a 12/12 h light/dark cycle (lights on at 07h00, considered as *Zeitgeber* time zero (ZT 0)) and received water and food *ad libitum*. The animals were killed by decapitation at ZT 6. All experiments were carried out in compliance with ethical standards of our institutional ethical committee (CEUA/IB-USP: license number 045/2007) and with the recommendation of the National Council on Experimental Animal Control (CONCEA).

### Drugs

Lipopolysaccharide (LPS, from *E. coli* serotype 0127:B8), penicillin/streptomycin, HEPES, BGJb medium and bovine albumin fraction V, minocycline hydrochloride, N-acetyl-leucinyl-leucinyl-norleucinal-H (ALLN), DL-fluorocitric acid barium salt, trypsin and trypsin inhibitor were obtained from Sigma (St Louis, MO, USA); Dulbecco’s Modified Eagle Medium (DMEM), and 6-diamidino-2-phenylindole (DAPI) were obtained from Invitrogen (Carlsbad, CA, USA); DL-fluorocitric acid barium salt was prepared as referenced [25]. Minocycline, ALLN and LPS were diluted directly in the medium.

### Organ Culture

Freshly-removed rat pineal glands were incubated (37°C, 95% O_2_, 5% CO_2_, 48 h) in BGJb medium enriched with 2 mM glutamine, 100 U/mL penicillin and 10 µg/mL streptomycin (pH 7.4) in a 24-multiwell plate (1 gland per well, 200 µL of medium per well), as previously described [26]. The medium was replaced every 24 h.

### Pinealocyte Culture

Primary pinealocyte cultures were prepared from rat pineal glands as previously described [27], with some modifications. Briefly, pinealocytes were obtained by trypsinization (0.25%, 37°C, 15 min) followed by mechanical dispersion in the presence of trypsin inhibitor (0.3%) in a solution containing mmol/L quantities of the following: 120 NaCl, 5 KCl, 25 NaHCO_3_, 1.2 KH_2_PO_4_, 12 glucose and 0.1% w/v bovine serum albumin. After centrifugation (15 min, 1000× g), the cells were resuspended in DMEM supplemented with 100 U/mL penicillin and 100 µg/mL streptomycin (pH 7.4). The total number of cells and fractional survival was estimated by Trypan blue exclusion. The survival rate was 90% or higher. Cells (0.7 × 10^5^) were seeded on poly-L-lysine coated 8-well chamber plate and maintained at 37°C, 5% CO_2_ for 18 h prior to experimental analysis.

### *In vitro* Treatments

The effect of LPS (0.1 µg/mL) on microglial activity and TNFR1 expression were determined in cultured pineal glands, and pinealocytes, respectively. The induction of TNF by LPS was determined both in gland and cell cultures. Microglial and astrocyte activity was blocked with minocycline (1 to 300 µM, 1 h) or fluorocitrate (1 to 300 µM, 1 h), respectively. The NF-kB pathway was blocked with ALLN (12.5 µM) 48 h prior to LPS stimulation of cultured pineal glands. The control for each experiment consisted of incubation with the vehicle solution of each drug used. The medium was stored for no longer than one month at –20°C before determining the concentration of TNF by ELISA.

**Figure 1:**
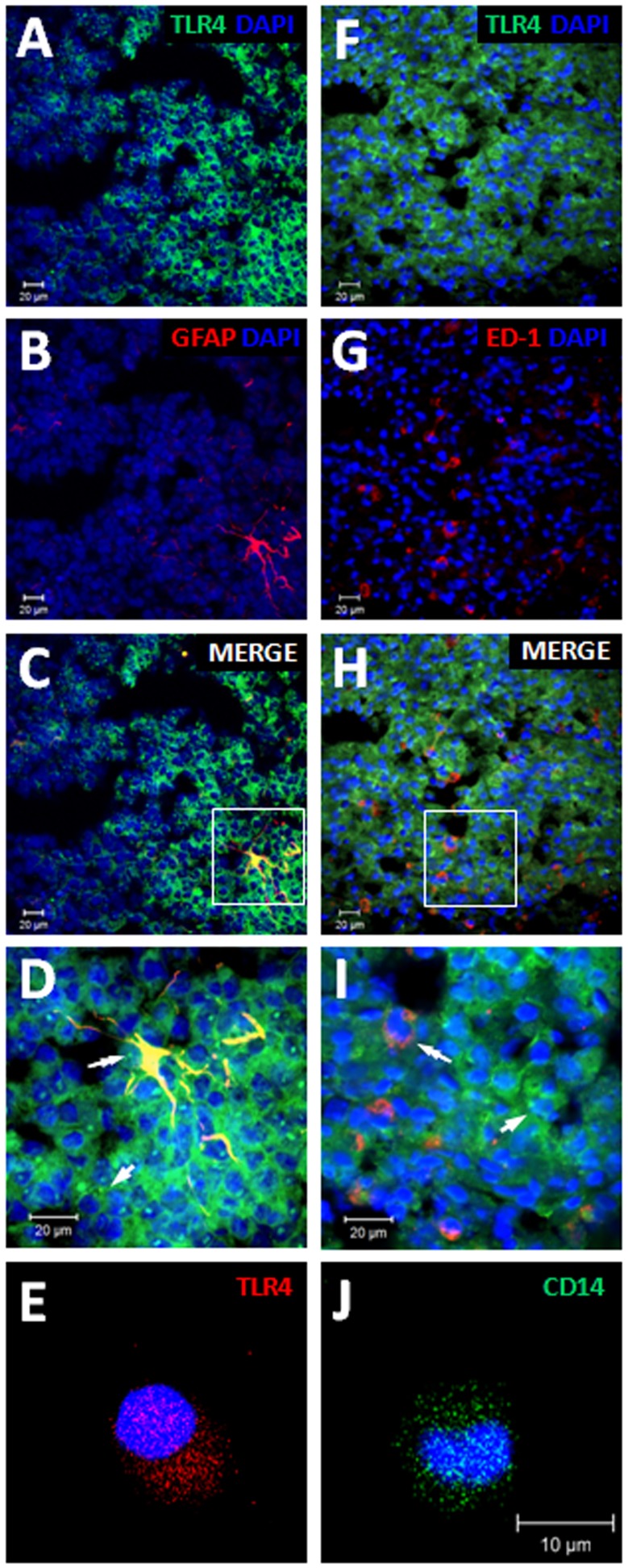
Cellular expression of TLR4 in the rat pineal gland. Representative images of TLR4 co-localization in astrocytes (A-D) and microglia (F-I). Green staining (A-E and F-I) represents immunoreactivity of TLR4. Red staining (B and G) represents immunoreactivity of GFAP (B) or ED-1 (G). Merged images (C, D, H and I) indicate co-localization of TLR4 and glia staining (double-arrow). As 90% of the pineal gland is composed by pinealocytes, the adjacent staining of TLR4 suggests expression in this cell type (single arrow). In order to confirm this result we performed immunocytochemistry assays and detected constitutive expression of TLR4 (E) and CD14 (J) in isolated pinealocytes. D and I correspond to the higher magnification of C and H, respectively (100 × objective). The nuclei were stained with DAPI (blue).

### Immunohistochemistry

The expression of TLR4 and double-labeling of TLR4 with ED-1 or GFAP were performed by immunohistochemistry assay as previously described [21] in frozen sections o pineal glands obtained from animals killed at ZT 6. Briefly, animals were anesthetized by intramuscular injection of ketamine (160 mg/kg) and xylazine (40 mg/kg) and perfused transcardially with 150 mL of saline followed by 300 mL of cold 4% paraformaldehyde, pH 9.5. Each pineal gland was removed from the skull and maintained at 4°C for 24 h in PBS plus 20% sucrose. Pineal glands were then embedded in medium for frozen tissue specimens (Tissue-Tek, Sakura Finetek, Torrance, CA, USA), frozen in dry ice and stored at –80°C till processing. Cryostat sections cut at a thickness of 20 µm were fixed in 4% paraformaldehyde (30 min, pH 9.5) and were incubated with 0.1 M glycine (5 min) followed by 1% albumin and 0.01% saponin in PBS (1 h, room temperature). Endogenous biotin was blocked using the Avidin-Biotin Blocking kit as suggested by the manufacturer (Vector Laboratories, SP2001, Burlingame, CA, USA). Rabbit polyclonal antibody anti-TLR4 (1∶200, Abcam, Cambridge, MA, USA) was incubated for 48 h at 4°C followed by incubation with an appropriate secondary antibody conjugated with FITC (1∶200, Sigma) for 1 h at room temperature. Next, the sections were incubated with 0.1 M glycine (5 min) followed by 1% albumin and 0.01% saponin in PBS (1 h, room temperature) and then incubated with appropriate antibodies for the identification of astrocytes (mouse monoclonal Cy3-conjugated GFAP, 1∶500, Sigma) or microglia (mouse monoclonal anti-ED-1, 1∶100, Abcam) followed by the appropriate secondary antibody conjugated with Cy3 (1∶200, Jackson ImmunoResearch, West Grove, PA, USA). All procedures were repeated at least 3 times to confirm the results. Images were acquired by a Confocal Laser-scanning microscope with a 40 × or 100 × objective and Zeiss LSM 510 (Zeiss confocal software, Germany). FITC was excited at 488 nm (Argon laser) and emitted fluorescence was measured at 515–530 nm. Cy3 was excited at 543/633 nm (HeNe laser) and emitted fluorescence was measured at 560 nm. An enterprise laser (excitation 364 nm and emission filter of 435–485 nm) was used for 4′, 6-diamidino-2-phenylindole (DAPI) imaging.

For microglial reactivity analysis, the pineal glands were cultured and incubated with LPS (0.1 µg/mL, 2 h). The cultured glands were fixed in 4% cold paraformaldehyde, pH 9.5 for 3 days at 4°C, followed by 24 h incubation in PBS plus 20% sucrose. Pineal glands were then frozen and the immunohistochemistry assay was performed as described previously by incubation with primary antibody (mouse monoclonal anti-ED-1, 1∶100, Abcam) followed by the secondary antibody conjugated with biotin (1∶200, Sigma). The peroxidase activity was revealed with 3, 3′-diaminobenzidine (DAB substrate kit for Peroxidase, Vector Laboratories). Photomicrographs were obtained from bright-field microscope (Nikon Eclipse E1000 coupled to a CoolSNAP-Pro Color digital camera) using the Image-Pro® Plus software (Media Cybernetics, Silver Spring, MD, USA).

The negative controls were performed by omission of the primary antibodies from the procedures and the substitution of normal serum from the same species. Staining was completely abolished under these conditions.

**Figure 2:**
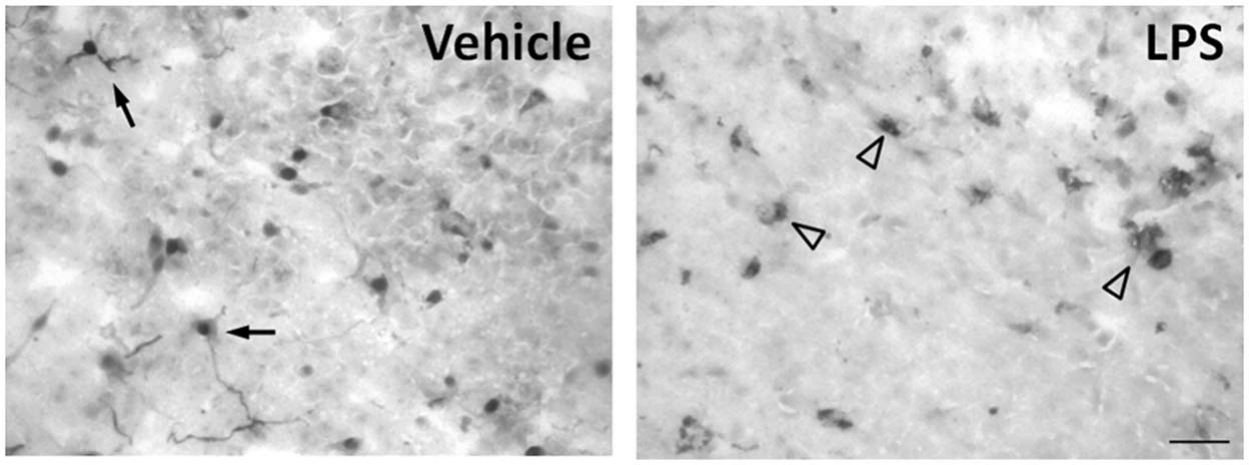
LPS induces microglia activation *in vitro*. Representative immunohistochemistry image of frozen pineal sections stained with ED-1 (CD68) antibody. In control tissue, microglial cells are present as small cellular bodies with long branched processes, as expected in a resting or surveillance state (single arrow). LPS changed the morphology of the microglia to cells with larger bodies and no branches, suggesting an activated state (arrow head). n  = 3–4 glands. The experiments were repeated twice.

### Immunocytochemistry

The immunocytochemistry assay was performed as previously described [21]. Briefly, cultured pinealocytes were fixed in 4% cold paraformaldehyde for 10 min and permeabilized with PBS plus saponin 0.5% at room temperature. The non-specific binding sites were blocked with 1% bovine serum albumin fraction V (BSA) and 0.3 M glycine for 60 min. The cells were then incubated with primary rabbit polyclonal antibody anti-TNFR1 (1∶500, Abcam), anti-CD14 (1∶200, Abcam) or anti-TLR4 (1∶500, Abcam) for 18 h at 4°C, followed by secondary polyclonal anti-rabbit conjugated with Texas Red antibody (1∶400, Abcam) or FITC (1∶200, Sigma) for 1 h at room temperature. Nuclei were stained with DAPI (300 µM, 5 min) at room temperature. Primary and secondary antibodies were diluted in blocking buffer. Images were acquired by a Confocal Laser-scanning microscope with a 40 × oil-immersion objective and Zeiss LSM 510 (Zeiss confocal software, Germany) using a HeNe 543/633 laser for Texas Red (excitation 590 nm; emission filter 650 nm), and enterprise laser at excitation 364 nm and emission filter of 435–485 nm for DAPI imaging. Fluorescence was quantified by Image J Software (http://rsb.info.nih.gov/ij).

The negative controls were performed by omission of the primary antibodies from the procedures and the substitution of normal serum from the same species. Staining was completely abolished under these conditions.

**Figure 3:**
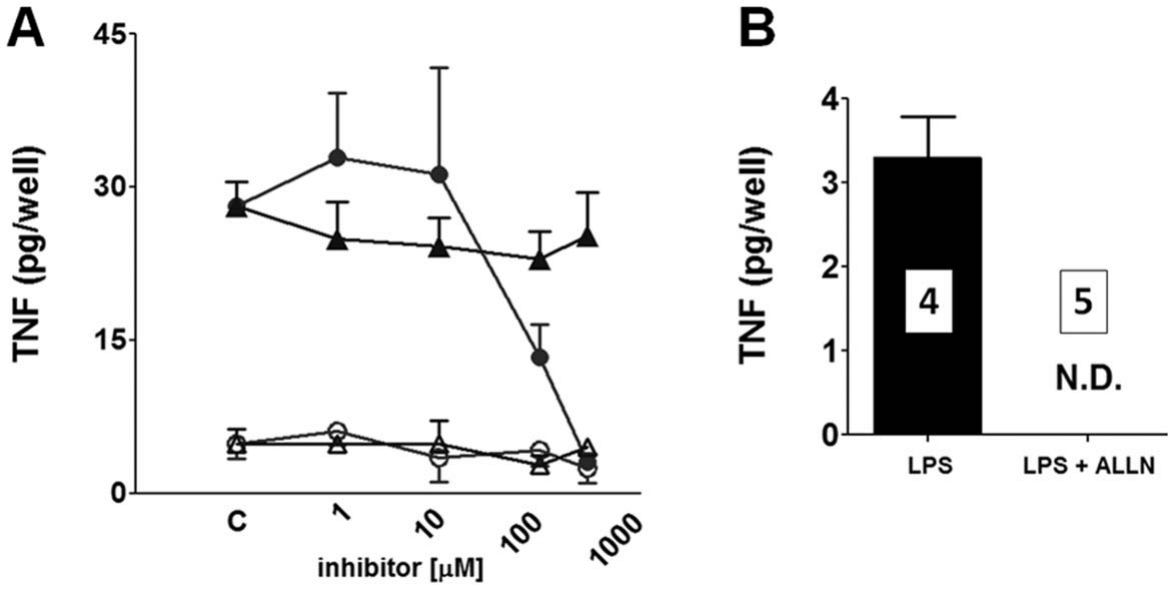
LPS induces TNF synthesis in cultured pineal glands dependent on microglia activation and NF-kB **activity.** A) The effect of blocking microglia (circles, minocycline, 1 to 300 µM, 1 h) or astrocyte activation (triangles, fluorocitrate, 1 to 300 µM, 1 h) on TNF levels. As observed, minocycline or fluorocitrate *per se* (open symbols) does not induce TNF production in cultured pineal glands. Values obtained in the absence of the inhibitors (control, C) are also plotted. Minocycline, but not fluorocitrate, blocks LPS-induced (closed symbols) TNF production in a concentration-dependent manner. These data indicate that microglial cells are the pivotal producers of TNF in the rat pineal gland. The data are expressed as the mean ± S.E.M. of 12–14 glands per point obtained in three different experiments. B) The effect of blocking the NF-kB pathway on TNF production. As observed, ALLN (12.5 µM, 48 h) fully abolished the LPS-induced TNF synthesis in cultured rat pineal gland. The data are expressed as the mean ± S.E.M., n  = 4–5 glands obtained in two different experiments. N.D.  =  not detected.

### TNF Detection

TNF concentration in the medium was measured with a commercially available rat TNF-alpha ELISA Ready-set-go kit (cat. 88-7340, eBioscience, San Diego, USA).

### Statistical Analysis

Data are presented as the mean ± S.E.M. Statistical analysis was performed using the student’s *t* test or ANOVA followed by Newman-Keuls test. Values of *P*<0.05 were considered statistically significant.

**Figure 4:**
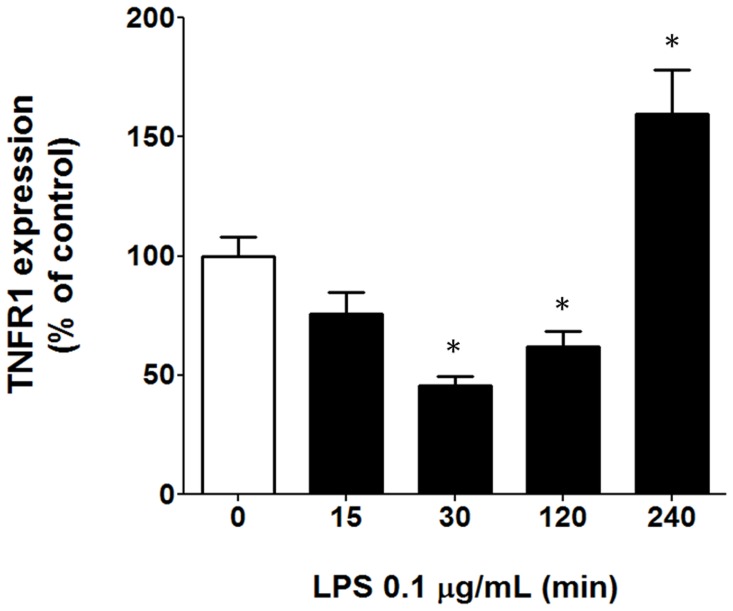
LPS modulates TNFR1 expression in pinealocytes. Quantitative analyses performed through ImageJ software demonstrated that LPS (0.1 µg/mL) induced the down-regulation of TNFR1 after 30 min and 2 h of incubation and up-regulation of TNFR1 after 4 h incubation. The data are expressed as the mean ± S.E.M. of the % relative to control samples from 80 cells obtained in 4 different experiments.

## Results

### Cellular Distribution of TLR4 in the Rat Pineal Gland

TLR4 expression was detected by immunohistochemistry in frozen pineal gland sections from rats perfused at ZT 6. TLR4-positive immunostaining was diffusely observed in the pineal parenchyma [21]. In order to evaluate the cell type that expressed TLR4 in the gland we double-labeled the receptor with glial markers or cultured isolated pinealocytes for immunocytochemistry. Here we used ED-1 (CD68, Cluster of Differentiation 68) or GFAP (Glial Fibrillary Acidic Protein) for double-staining TLR4 and microglia or astrocytes, respectively. TLR4-positive immunostaining co-localized with both astrocyte (GFAP, Fig. 1A-D) and microglia (ED-1, Fig. 1F-I) markers, and was present on isolated pinealocytes (Fig. 1E), indicating that these three cell types present in the pineal gland express TLR4 and are able to respond to LPS. We also detected constitutive expression of CD14 (Cluster of Differentiation 14) in isolated pinealocytes (Fig. 1J).

**Figure 5:**
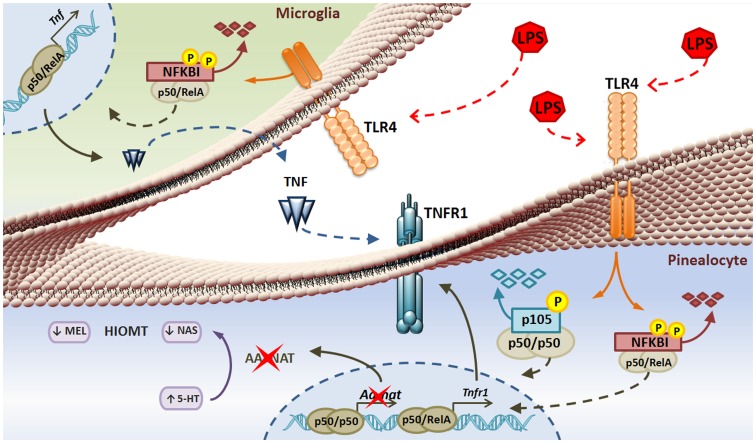
Microglial cells mediate LPS-induced TNF production in the rat pineal gland through NF-κB signaling and suppress melatonin synthesis in a paracrine manner. LPS activation of TLR4 is translated through phosphorylation and degradation of the nuclear factor kappa B inhibitor (NFKBI) allowing the nuclear translocation of both gene transcription repressor (p50/p50) and activator (p50/RelA) NF-kB forms a dimer in pinealocytes and microglia. In microglia, LPS induces TNF production that may activate TNFR1 expressed on pinealocytes. In addition, LPS up-regulates TNFR1 expression, most probably to allow maximal TNF signaling. Therefore, melatonin (MEL) and N-acetylserotonin (NAS) production is suppressed due to the repressive activity of the p50/p50 NF-kB dimer that may form due to TNF downstream signaling.

### Microglia Mediate LPS-induced TNF Production

Microglial reactivity was evaluated by immunohistochemistry in frozen sections of cultured pineal glands stimulated with LPS (0.1 µg/mL, 2 h) and stained with ED-1. Microglial cells were diffusely distributed in the pineal parenchyma (Fig. 2). In control glands, microglial cells were present as small cellular bodies with long branched processes, as expected in a resting or surveillance state. In LPS-treated pineal glands, the microglia cells present larger bodies and no branches suggestive to an activated state [28].

Isolated pinealocytes incubated with LPS (0.1 µg/mL, 0 to 6 h) did not produce detectable amounts of TNF in the medium, suggesting that other cell types produce this cytokine. The inhibition of microglia by minocycline, but not of astrocytes by fluorocitrate, inhibited LPS-induced pineal gland synthesis of TNF, indicating that microglia were the major cell type that synthesized this cytokine (Fig. 3A).

As seen in other immune defense cells [29], TNF production in the cultured pineal glands was mediated by activation of the NF-kB pathway. LPS-induced TNF production was blocked by ALLN, a classical inhibitor of proteasomes (Fig. 3B).

### LPS Regulates TNFR1 Expression in Isolated Pinealocytes

The time-course of the LPS effect on TNFR1 expression on isolated pinealocytes was determined after 15, 30, 120 and 240 min of stimulation. LPS induced a bimodal regulation of TNFR1 expression. Between 30 and 120 min, a down regulation was observed, while in the interval between 120 and 240 min there was an up-regulation (Fig. 4).

## Discussion

The pineal gland, besides being the neuro-endocrine transducer of daily dark/light cycles, also plays a relevant role in regulating immune responses. Besides producing melatonin, the pineal gland can also be induced to synthesize several cytokines, such as such as transforming growth factor-beta1, interleukin-6, interleukin-1-beta and TNF [21], [30]–[32]. The understanding of the relationship between the synthesis of melatonin and cytokines by pineal glands challenged with danger signals is in the center of the questions regarding the role of pineal gland in an innate immune response. The present paper focused on demonstrating that the microglia present in the pineal gland, instead of astrocytes or even pinealocytes are the cells that answer to LPS for synthesizing TNF.

A rise in nocturnal melatonin is one of the mechanisms that inhibit leukocyte migration to healthy tissues [33]. Reduction of nocturnal plasma melatonin, a prerequisite for the proper induction of an inflammatory response, is based on the ability of the pineal gland to detect and respond to danger signals. We have previously shown that intravenous LPS injection significantly reduces plasma melatonin at night, and increases the expression of adhesion molecules by endothelial cells [34]. In addition, LPS reduces noradrenaline-induced melatonin synthesis by triggering the NF-kB pathway through activation of TLR4 [21].

Besides modulating melatonin synthesis, LPS triggers the pineal synthesis of TNF, detectable in the culture medium after 2 h and attaining a maximal concentration after 4 h of incubation [21]. We observed that pinealocytes, astrocytes and microglia expressed TLR4, and therefore could be directly activated by LPS, as suggested by the activation of microglia after intravenous injection of LPS [35] or by *in vitro* incubation (present work). Other circumventricular organs, such as the organum vasculosum of the lamina terminalis, area postrema, choroid plexus, meninges, subfornincal organ and median eminence also express TLR4 [36], [37]. Therefore, besides the pinealocytes, microglia and astrocytes are also able to respond to LPS, raising the question of whether TNF is synthesized by one specific cell type.

As isolated pinealocytes were not able to synthesize detectable amounts of TNF, we focused on the glial cells. The pharmacological inhibition of astrocytes or microglia indicated that only pineal gland microglial cells were able to produce TNF. This correlated with observed effects of LPS administration in the central nervous system, as the rolling and adhesion of leukocytes and TNF production are blocked by minocycline [38], an inhibitor of microglia activation that inhibits TNF synthesis in several areas of the central nervous system. Although astrocytes are known to synthesize TNF when stimulated with LPS, our data show that under the experimental conditions of the present study, no TNF is produced by pineal gland astrocytes.

TLR4 signal transduction occurs mainly through NF-kB, an important downstream signaling pathway for controlling physiological and pathophysiological conditions [39]–[41] and a master modulator of cytokine production [42]. NF-kB acts as either a repressor or inductor of gene transcription. Usually the homodimer p50/p50, which lacks a transactivating domain, represses gene transcription while the heterodimer p50/RelA induces gene transcription [43]. We have previously demonstrated that LPS induced the nuclear translocation of p50/p50 and p50/RelA dimers in the rat pineal gland [21]. As melatonin is synthesized by pinealocytes, and these cells express TLR4 we concluded that activation of the NF-kB pathway in pinealocytes inhibited melatonin production. This inhibition is probably mediated by the p50/p50 homodimer, while TNF production is dependent on the activation of the p50/RelA heterodimer in microglia, as it is inhibited by minocycline and ALLN. In summary, we concluded that LPS acts on pinealocytes and microglia, impairing the production of melatonin, and inducing the synthesis of TNF, respectively.

As activation of TNFR1 impairs noradrenaline-induced melatonin synthesis [22], [24] this strongly suggests that TNF produced by microglia amplifies the LPS-effect mediated by activation of TLR4 on pinealocytes. It is interesting to note that the peak of TNF production by microglia [21] and the maximal TNFR1 expression attained in pinealocytes (present paper) occurred 240 min after incubation of pineal glands with LPS. This fine-tuning between the timing of agonist production and expression of the receptor suggests that the amplification of the LPS effect by TNF represents a second activation of the nuclear translocation of NF-kB. Reinforcing the idea of this strict control in the timing of the response, LPS initially induced a reduction in pinealocyte expression of TNFR1 (from 30 to 120 min). It is interesting to note that in mouse bone marrow granulocytes, receptor shedding induced by LPS reduced the expression of TNFR1 over a similar time-course (20 min), by a p38 mitogen-activated protein kinase dependent mechanism [44], a signaling cascade known to participate in control of the amplitude and duration of the nocturnal peak of the key enzyme in melatonin synthesis [45]. Therefore, the initial reduction of TNFR1 receptors also favors the idea that the peak of NF-kB activation in pinealocytes induced by TNF is programmed to occur after the initial wave triggered by direct LPS activation of TLR4 [29], [46].

The relevance of pineal production of TNF is currently unclear, although TNF can inhibit noradrenaline-induced transcription of AA-NAT and the synthesis of N-acetylserotonin [22], [24]. This mechanism is also important for controlling the nocturnal melatonin surge in humans. The suppression of the nocturnal melatonin surge in innate immune responses to mastitis [11] or surgery incision [12] is inversely correlated with TNF. In addition, it was observed that the return of nocturnal melatonin pineal output was strictly dependent upon the conclusion of the TNF peak [30].

In conclusion, (Fig. 5), the pineal gland is a target for LPS, as TLR4 is expressed on pinealocytes, astrocytes and microglia. The effect of LPS on pinealocytes, the effector cells of the pineal gland, occur not only by direct stimulation of TLR4, but also by inducing TNF production in microglia. In addition, the expression of TNFR1 on pinealocytes is under fine-tuned temporal control. Finally, the complexity of LPS effects on the pineal gland provides the first insight into strategies that can ensure proper control of the expression of circadian timing during the evolution of an innate immune response.
